# The expression of MMP‐1 and MMP‐9 is up‐regulated by smooth muscle cells after their cross‐talk with macrophages in high glucose conditions

**DOI:** 10.1111/jcmm.13728

**Published:** 2018-07-10

**Authors:** Razvan Daniel Macarie, Mihaela Vadana, Letitia Ciortan, Monica M. Tucureanu, Andrea Ciobanu, Dragos Vinereanu, Ileana Manduteanu, Maya Simionescu, Elena Butoi

**Affiliations:** ^1^ Institute of Cellular Biology and Pathology “Nicolae Simionescu”, Biopathology and Therapy of Inflammation Bucharest Romania; ^2^ Cardiology Department Carol Davila University of Medicine and Pharmacy, University and Emergency Hospital Bucharest Bucharest Romania

**Keywords:** atherosclerosis, cell cross‐talk, high glucose, matrix metalloproteinases, protein kinase C

## Abstract

Patients with diabetes mellitus have an increased risk of myocardial infarction and coronary artery disease‐related death, exhibiting highly vulnerable plaques. Many studies have highlighted the major role of macrophages (MAC) and smooth muscle cells (SMC) and the essential part of metalloproteases (MMPs) in atherosclerotic plaque vulnerability. We hypothesize that in diabetes, the interplay between MAC and SMC in high glucose conditions may modify the expression of MMPs involved in plaque vulnerability. The SMC‐MAC cross‐talk was achieved using trans‐well chambers, where human SMC were grown at the bottom and human MAC in the upper chamber in normal (NG) or high (HG) glucose concentration. After cross‐talk, the conditioned media and cells were isolated and investigated for the expression of MMPs, MCP‐1 and signalling molecules. We found that upon cross‐talk with MAC in HG, SMC exhibit: (*i*) augmented expression of MMP‐1 and MMP‐9; (*ii*) significant increase in the enzymatic activity of MMP‐9; (*iii*) higher levels of soluble MCP‐1 chemokine which is functionally active and involved in MMPs up‐regulation; (*iv*) activated PKCα signalling pathway which, together with NF‐kB are responsible for MMP‐1 and MMP‐9 up‐regulation, and (*v*) impaired function of collagen assembly. Taken together, our data indicate that MCP‐1 released by cell cross‐talk in diabetic conditions binds to CCR2 and triggers MMP‐1 and MMP‐9 over‐expression and activity, features that could explain the high vulnerability of atherosclerotic plaque found at diabetic patients.

## 1 INTRODUCTION

Type 2 diabetes mellitus (T2DM) is a recognized risk factor for cardiovascular morbidity and mortality.^1^ Patients with T2DM have an increased risk of premature atherosclerosis, heart failure, peripheral arterial insufficiency and microvascular complications, affecting life quality and expectancy.^2^, ^3^

Diabetes (ie hyperglycemia) is one of the major determinants for atherosclerotic plaque progression towards vulnerability. As reported, the atherosclerotic plaque is a dynamic structure, resulting from an interplay between different cellular and humoural factors, as well as extracellular matrix (ECM) component consisting largely in type I collagen and proteoglycans. A key event in the progression of the atherosclerotic plaque towards a vulnerable one is the alteration of the ECM composition. The remodelling of ECM is controlled mainly by matrix MMPs that are produced within the plaque by MAC and SMC. The MMPs belong to a large family of zinc dependent endopeptidases that degrade almost all ECM components in both physiological and pathological processes.^4^ By degrading the native collagen, the MMPs lead to low resistance of the vessel to mechanical or other stresses,^5^ digest the ECM of the fibrous cap, inducing the cap thinning and ultimately, plaque rupture. Therefore, the MMPs are actively involved in atherosclerosis by both, promoting migration and proliferation of SMC, and induction of ECM remodelling and plaque destabilization.^6^

The data on the involvement of gelatinases and collagenases in the fibrous cap weakening came from histological analysis of sections from rabbit and mouse atherosclerotic plaques and from rupture‐prone shoulder regions of human atherosclerotic plaques which showed increased levels of MMP‐1, MMP‐3 and MMP‐9.^7^, ^8^ Recently, we found that the expression of MMP‐1 and MMP‐9 is increased by cross‐talk between the MAC and SMC.^9^

To date, there are studies suggesting that the atherosclerotic plaques of the subjects with T2DM are more prone to rupture because of both, increased vascular inflammation,^10^ and impaired repair responses,^11^ as loss of collagen and other ECM components occurs in the highly inflamed regions of plaque cap. However, the exact players involved in exacerbation of plaque vulnerability in diabetic patients are not known.

It was also reported that MCP‐1 ‐ a small chemoattractant protein with an established role in inflammation of atherosclerosis ‐ binds to its main receptor, CCR2, inducing cell signalling and stimulating the migration of immune cells to the sites of infection or inflammation.^12^ There is overwhelming evidence that MCP‐1 plays a critical role in diabetes and ensuing cardiovascular disorders. To date, the increased level of serum MCP‐1 correlated with different markers of type 2 diabetes and obesity,^13^ and CCR2 expression on monocytes was found to be higher in diabetic patients.^14^ Circulating levels of MCP‐1 are higher in patients with acute myocardial infarction, ischaemic stroke and unstable angina, but not in patients with stable angina, suggesting the involvement of MCP‐1 in plaque vulnerability.^15^, ^16^

In the last decade, we and others found that the interaction(s) between SMC and monocytes/MAC produces different inflammatory molecules including MCP‐1,^17^ leading to monocyte/macrophage activation, the switch of SMC towards a synthetic phenotype,^18^, ^19^, ^20^ and a reduction of collagen I and III expression.^9^ The effect of HG (ie diabetes) on MAC‐SMC cross‐talk and whether this is related to accelerated plaque evolution in diabetic patients is not known.

In this context, we hypothesized that the molecular signals resulted from the cross‐talk between SMC‐MAC in HG conditions may increase the synthesis of main MMPs involved in atherosclerotic plaque thinning, changes that would tilt the balance towards the vulnerable plaque. To this purpose, we designed experiments to investigate the effect of cross‐talk between MAC and SMC in HG conditions (associated to diabetes mellitus) on the key MMPs known as ECM destructors, and on the mechanisms involved in MMPs modulation.

We provide evidence that in SMC, upon communication between SMC‐MAC in HG conditions increases the expression and activity of MMP‐1 and ‐9 by a mechanism involving the MCP‐1/ CCR2 and PKC signalling pathway. The data could explain the enhanced propensity of the atherosclerotic plaque for rupture and the ensuing athero‐thrombosis in diabetic patients and provide new therapeutic targets for this disease.

## 2 MATERIAL AND METHODS

### 2.1 Chemicals

SiRNAs (CCR2/p65/scrambled) were from Santa Cruz Biotechnology and siRNA transfection reagent Hiperfect were obtained from Qiagen. RT‐PCR reagents and enzymes were from Invitrogen. Monoclonal antibodies, anti‐human MMP‐1 (MAB901), anti‐human CCR2 (MAB180) were from R&D Systems, UK, anti‐MMP‐9 (ab76003) from Abcam, anti‐phospho‐PKC (A‐11) from Santa Cruz and anti‐actin (A5060) from Sigma‐Aldrich. Human MCP‐1 ELISA kit (DuoSet) and Go 6976 (PKC inhibitor) were from R&D Systems. All other substances and materials were from Sigma‐Aldrich, Invitrogen, Life Technologies, Eppendorf, Bio‐Rad.

### 2.2 Cell culture

Human aortic SMC were isolated from the media of foetal thoracic aorta and characterized as a pure cell line devoid of any contaminants. The cells exhibited an elongated spindle‐shaped morphology, bundles of cytoplasmic myofilaments and numerous caveolae at the cell periphery (as demonstrated by electron microscopy), and grow as multilayers with the characteristic hills and valley pattern (as assessed by phase‐contrast microscopy). In addition, immunoblotting and immuno‐histochemistry experiments revealed that the cells were positive for smooth muscle alpha‐actin, and for vinculin, negative for von Willebrand factor,^21^ and displayed functional store‐operated channels responsive for capacitative calcium entry. SMC were cultured in DMEM supplemented with 10% FCS.

Monocyte‐like cell line THP‐1 were grown in suspension in the RPMI 1640 culture medium containing 10% FCS and were split 1:5, twice a week.

Blood human monocytes were isolated from healthy donors (from Blood Transfusion Center) or from diabetic patients with aortic coronary syndrome (ACS) (from the Cardiology Clinic, University Emergency University Hospital, Bucharest) using a Ficoll gradient and cultured in RPMI 1640 containing 10% FCS. The investigation was carried out according to the principles outlined in the Declaration of Helsinki^22^ for experiments involving human samples. The participants gave their written informed consent by signing the appropriate paperwork and respecting their anonymity and privacy rights. The Ethics Committee of the Institute of Cellular Biology and Pathology “Nicolae Simionescu,” Bucharest, and of the University Emergency Hospital have approved the study.

### 2.3 Experimental design: the co‐culture system

Human SMC (5 × 10^4^ cells/well) were grown to confluence on the bottom of Transwell co‐cultures chambers.

Human monocytes (1 × 10^6^ cells) were plated on the filter inserts (Falcon, 0.4 μm pore diameter) in RPMI containing high 25 mmol/L glucose (HG) or normal 5 mmol/L glucose (NG ‐ controls) and differentiated into MAC by exposure to 100 nmol/L PMA (THP monocytes) or 50 ng/mL M‐CSF (freshly isolated monocytes). After 3 days, the co‐culture was constructed by placing the inserts with MAC on the top of the Transwell co‐culture chambers. The co‐culture was kept in RPMI without serum, in normal (5 mmol/L glucose) or HG (25 mmol/L glucose) concentration for 24 or 72 hours. For every experiment, differentiated macrophages (M) or confluent SMC (S, not co‐cultured), maintained independently in the serum free medium and NG concentration, were used as control. At the end of cell co‐culture time, the conditioned media of MAC (upper chamber) or conditioned media of SMC (bottom chamber) were collected and used for further determination (MCP‐1 ELISA and gelatin zymography assays), and the cells (MAC from upper chamber and SMC from bottom chamber) were subjected to different investigation methods, as described below.

### 2.4 Western Blot

Protein expression of MMP‐1, MMP‐9, pPKC, CCR2, p65 and actin were assessed in the total extract of SMC obtained after cell homogenization in Laemmli electrophoresis buffer as described.^9^ The signals were visualized using SuperSignal West Pico chemiluminescent substrate (Pierce) and quantified by densitometry employing gel analyzer system Luminiscent image analyzer LAS 4000 (Fujifilm) and Image reader LAS 4000 software.

### 2.5 Transfection of small‐interfering RNA (siRNA)

Human siRNAs (CCR2, p65 subunit/scrambled) were transfected into human SMC at 24 hours after their passage using siRNA transfection reagent Hiperfect^®^ (Qiagen) according to the manufacturer's protocol. Twenty‐four hours after transfection, the cells were incubated with serum free medium and co‐cultured with PMA‐differentiated macrophages, for another 24 hours. Then, the SMC were processed for further protocols, as described.

### 2.6 Real‐time PCR

Total RNA was isolated from cells using GenElute^®^ Mammalian Total RNA kit (Sigma)/or trizol. First‐strand cDNA synthesis was performed employing 1 μg of total RNA and MMLV reverse transcriptase, according to the manufacturer's protocol (Invitrogen). Assessment of mRNA expression for different molecules was done by amplification of cDNA using a LightCycler 480 Real Time PCR System (Roche) and SYBR Green I chemistry. The primer sequences are shown in Table 1. Optimized amplification conditions were: 0.2 mmol/L of each primer, 2.5 mmol/L MgCl_2_, annealing at 60°C and extension at 72°C for 40 cycles. The relative quantification was done by comparative CT method and expressed as arbitrary units. The beta‐actin was used as reporter gene for all the investigated molecules.

**Table 1:** The sequences of oligonucleotide primers used for evaluation of gene expression

Gene	GenBank^®^ accession number	Sequences of Oligonucleotide Primers	Predicted size (bp)
MMP‐1	NM_002421.3	Fw: 5′‐aaaattacacgccagatttgcc‐3′ Rv: 5′‐ggtgtgacattactccagagttg‐3′	82
MMP‐2	NM_004530.5	Fw: 5′‐ccgtcgcccatcatcaagtt‐3′ Rv: 5′‐ctgtctggggcagtccaaag‐3′	169
MMP‐9	NM_004994.2	Fw: 5′‐gtgcgtcttccccttcactttcct‐3′ Rv: 5′‐ggaatgatctaagcccagcg‐3′	199
MMP‐13	NM_002427.3	Fw: 5′‐actgagaggctccgagaaatg‐3′ Rv: 5′‐gaaccccgcatcttggctt‐3′	103
TIMP‐1	NM_003254.2	Fw: 5′‐aattccgacctcgtcatcag‐3′ Rv: 5′‐tgcagttttccagcaatgag‐3′	230
TIMP‐2	NM_003255.4	Fw: 5′‐atgcaggtggattccttcag‐3′ Rv: 5′‐aaacgatgccaaatggagag‐3′	306
β‐actin	NM_001101.4	Fw: 5′‐catgtacgttgctatccaggc‐3′ Rv: 5′‐ctccttaatgtcacgcacgat‐3′	250

### 2.7 MCP‐1 quantification by ELISA assay

Soluble MCP‐1 levels were quantified in the conditioned media of MAC or SMC collected after cell cross‐talk (as described in the “Experimental design: the co‐culture system” section), using an ELISA DuoSet kit (R&D systems) which measure MCP‐1 in the cell culture supernatants, serum and plasma, according to the manufacturer's instructions.

### 2.8 Gelatin zymography assay

The gelatinolytic activity of MMP‐2 and MMP‐9 was evaluated by gelatine zymography, as we described previously.^18^ Briefly, conditioned media collected from cells exposed to different experimental conditions were centrifuged at 800 *g*, mixed with non‐reducing Laemmli sample buffer and subjected to electrophoresis under non‐reducing conditions on 10% polyacrylamide gels containing 1 mg/mL gelatine, as substrate. After electrophoresis, the gels were re‐natured in 2.5% Triton X‐100 (2 × 30 minutes) and then incubated with 50 mmol/L Tris‐HCl, pH 7.4, containing 10 mmol/L CaCl_2_ and 0.2 mmol/L PMSF (18 hours, 37°C); subsequently the gels were stained with 0.2% Coomassie brilliant blue R‐250 and de‐stained with 10% acetic acid and 25% methanol. The white bands against the blue background were indicative for the presence of gelatinolytic activity. Image acquisition was done with a gel analyzer system Luminiscent image analyzer LAS 4000 (Fujifilm).

### 2.9 Assessment of SMC‐mediated collagen assembly

In a first step, the collagen Type I obtained from rat tail (powder from Sigma) was labelled with Texas Red as previously described.^23^ Briefly, collagen was solubilized in 2 mmol/L HCl to a final concentration of 10 mg/mL on a shaker at room temperature. Then, the collagen solution was brought to a basic pH of 8.3 necessary for labelling, using a solution of 0.1 mol/L Na_2_CO_3_ and 0.5 mol/L NaCl. At this pH the collagen solution gelled and the Texas Red dye (Jena Biocience, 10 mg/mL Texas Red Maleimide in DMF) was added. The labelling mixture was allowed to react for 60 minutes at room temperature, protected from light, with gentle shaking. The labelled protein solution was precipitated using a salt solution (0.9 mol/L NaCl) and centrifuged for 10 minutes at 7500 *g*. The Texas Red‐labelled collagen was then dialyzed against 0.2% acetic acid for 3 days at 4°C to remove any unbound dye and restore the acidic pH. Following dialysis, the gelled collagen was centrifuged again for 10 minutes at 7500 *g* and re‐solubilized in 200 μL acetic acid 0.2%.

In the second step, Texas Red‐labelled collagen was mixed with cold DMEM containing 10% foetal bovine serum to a final concentration of 5 μg/mL, concentration at which self‐assembly of collagen fibres was not observed. This mixture was added to cultured SMC that were previously co‐cultured for 24 hours with MAC in normal or HG conditions. After 24 hours at 37°C. SMC were washed with PBS, fixed with 4% paraformaldehyde for 20 minutes, counter‐stained with Hoechst 33259 and visualized with an inverted fluorescence microscope Olympus IX81.

### 2.10 Statistical analysis

The data obtained from all experiments are expressed as average ± standard deviation (SD). Statistical differences between 2 independent experimental groups were determined by using one‐way ANOVA and Tukey's multiple comparisons test from GraphPad Prism 7 software. Each set of experiments was performed at least 3 times. Values of *P* < .05 were considered significant. The “n” in the figure legends indicate the number of independent experiments.

## 3 RESULTS

### 3.1 Co‐culture of MAC and SMC in HG conditions increases the gene and protein expression of MMP‐1 and MMP‐9 in SMC

Since we previously found that the expression of MMP‐1 and MMP‐9 are increased subsequent to MAC‐SMC cross talk,^9^ we questioned now whether HG (ie diabetes) has an effect on this interaction. Thus, SMC and MAC were co‐cultured in NG or in HG conditions and the gene expression of collagenases (MMP‐1 and MMP‐13) and gelatinases (MMP‐2 and MMP‐9) were investigated in SMC.

**Figure 1:**
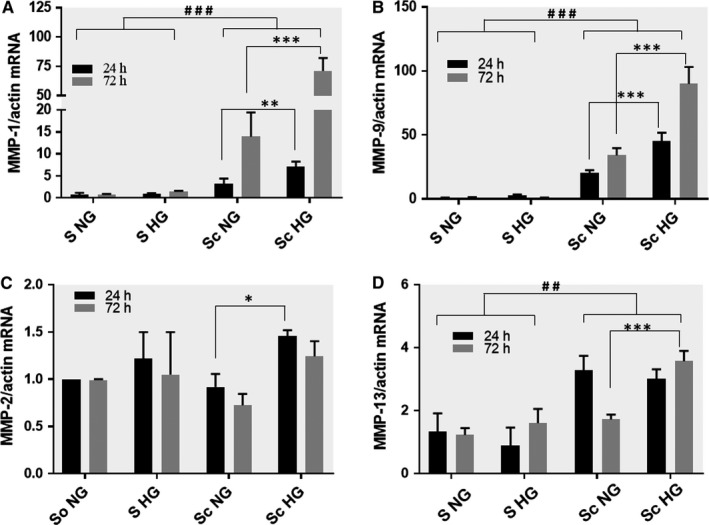
Gene expression of MMP‐1 (A), MMP‐9 (B), MMP‐2 (C) and MMP‐13 (D) in smooth muscle cells (SMC) subsequent to their co‐culture with macrophages (MAC) in normal and high‐glucose conditions. After 24 or 72 h, mRNA was obtained from cell homogenates and subjected to RT‐PCR. The mRNA of MMPs were normalized to actin mRNA. S NG, control SMC grown in normal glucose (5 mmol/L) concentration; S HG – SMC exposed to high (25 mmol/L) glucose concentration in the culture medium; Sc NG ‐ SMC after their co‐culture with MAC in normal glucose, Sc HG ‐ SMC after their co‐culture with MAC in high glucose conditions. Note the significant increase of MMP‐1 and MMP‐9 gene expression in SMC after their co‐culture with MAC in HG conditions compared to SMC co‐cultured with MAC in normal glucose or controls (SMC not co‐cultured, exposed to NG or HG conditions) at both, 24 or 72 h. n = 4, *P < .05, **P < .01, ***P < .001, ^##^ *P* < .01, ^###^ *P* < .001

The MMPs expression in SMC after their cross‐talk in HG conditions were compared with either, MMPs expression in the SMC after cross‐talk with MAC in NG conditions and in control cells ‐ consisting in SMC grown independently in NG or HG conditions, (as described in the experimental design). The quantitative PCR results showed that, compared to controls, the interaction between SMC and MAC in NG conditions for 24 or 72 hours, induced a significant increase in the expression of MMP‐1 and MMP‐9 in SMC (Figure 1A,B). Moreover, when the cells were co‐cultured in HG conditions, the MMP‐1 and MMP‐9 gene expression were further increased, suggesting that HG concentration exacerbate the cross‐talk induced expression of these MMPs.

The MMP‐2 gene expression was significantly increased in SMC after 24 hours co‐culture with MAC in HG compared with NG (Figure 1C). In the case of MMP‐13, the gene expression of this collagenase was significantly increased in SMC upon 24 or 72 hours co‐culture with MAC in HG concentration (Figure 1D). Compared with NG, the cell co‐culture in HG induces significantly increase of MMP‐13 only at 72 hours.

Exposure of SMC to HG (no co‐culture) did not affect the gene expression of investigated MMPs.

**Figure 2:**
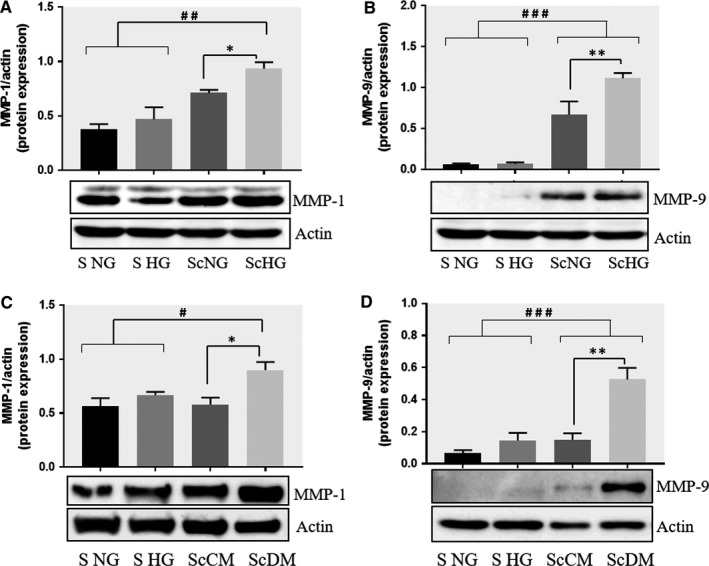
Co‐culture of SMC and MAC in high glucose conditions (24 h) induces a significant increase in the protein expression of MMP‐1 and MMP‐9. A, B: Protein expression of MMP‐1 (A) and MMP‐9 (B) was assessed in SMC after co‐culture with THP‐derived macrophages and evaluated by Western blot assay. C, D: Protein expression of MMP‐1 (C) and MMP‐9 (D) in SMC after co‐culture with human macrophages derived from freshly isolated monocytes from control subjects (CM) or diabetic patients with ACS (DM). S NG –control SMC, S HG – SMC exposed to HG, Sc NG or Sc CM – SMC which were co‐cultured with MAC in normal glucose, Sc HG or Sc DM ‐ SMC which were co‐cultured with MAC in high glucose. n = 3, *P < .05, **P < .01, ***P < .001, ^#^ *P* < .05, ^##^ *P* < .01, ^###^ *P* < .001

The data on the gene expression correlated well with the results obtained for protein expression of MMP‐1 and ‐9. As shown in the Figure 2A,B, the Western Blot results revealed a significant increase in protein level of MMP‐1 and MMP‐9 in SMC after co‐culture with MAC in HG conditions. The protein expression of MMP‐2 or MMP‐13 was not significantly modified by cell co‐cultured in HG compared with NG conditions (data not shown).

To validate these results, we performed similar experiments where, instead of using MAC derived from THP line, we have used human MAC‐derived from monocytes freshly isolated from the blood of healthy donors (control MAC, CM) or from diabetic patients with ACS and unstable plaque (diabetic MAC, DM). As shown in the Figure 2C,D, the MMP‐1 and MMP‐9 protein expression were significantly increased in SMC after co‐culture with MAC derived from monocytes freshly isolated from diabetic patients (ScDM) compared with control MAC (ScCM). To clearly delineate between the effect of diabetic MAC or HG on MMPs produced by SMC after co‐culture, both, the MAC derived from healthy donors (CM) or from diabetic patients (DM), were co‐cultured in normal or HG conditions with SMC. In these conditions, the MMP‐9 was induced in SMC co‐cultured with CM in HG compared with NG, and also in SMC co‐cultured with DM in HG compared with NG, suggesting the HG effect on MMP‐9 expression (Figure S1B). In addition, MMP‐9 expression is higher in SMC which were co‐cultured in HG with DM vs CM, indicating that even diabetic MAC are responsible for increased MMP‐9 expression. In case of MMP‐1, that main responsible for its up‐regulation is HG concentration, since no significant differences were obtained between the MMP‐1 expression in SMC co‐cultured with CM or DM in when they were exposed to HG conditions (ScCM HG vs ScDM HG, Figure S1A). The data corroborate well and confirm the results obtained with THP monocyte line.

### 3.2 Assessment of the gelatinases activity in the secretome collected from SMC after co‐culture with MAC in HG conditions

Since the activation state of gelatinases seems to be of crucial importance for their function in atherosclerosis, we questioned whether the MMP‐2 and MMP‐9 produced by the SMC after co‐culture with MAC are secreted and have gelatinolytic activity. To this purpose, we performed zymography experiments using the secretome (conditioned media) collected from SMC co‐cultured with MAC in HG or NG and analysed the MMP‐2 and MMP‐9 activity. As controls, individually cultured SMC grown in NG or in HG conditions were employed.

**Figure 3:**
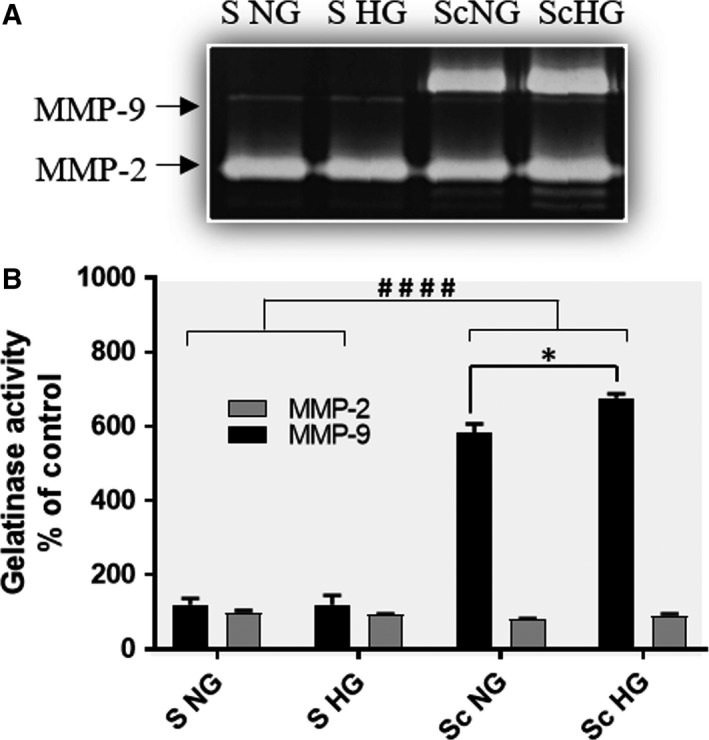
The enzymatic activity of MMP‐2 and MMP‐9 gelatinases assessed by SDS‐PAGE zymography of the conditioned media collected from SMC that were previously co‐cultured with MAC in normal or high glucose (Sc NG, Sc HG) conditions and from control cells (S NG or S HG). Note that the enzymatic activity of MMP‐9 released by SMC after cross‐talk with MAC is significantly increased in HG compared with NG. n = 4, *P < .05; ^####^ *P* < .001

As shown in Figure 3, the active MMP‐9 was detected in the SMC secretome collected after their co‐cultured with MAC, but not from that collected from control SMC, at least to a detectable level by zymography assay. Notably, the co‐culture of SMC and MAC in NG induces secretion of active MMP‐9 in medium isolated from SMC (Sc ng, Figure 3), which is additionally increased in the secretome isolated from SMC co‐cultured in HG conditions (Sc HG, Figure 3).

### 3.3 Effect of cellular cross‐talk in HG conditions on MCP‐1 production by SMC and MAC

**Figure 4:**
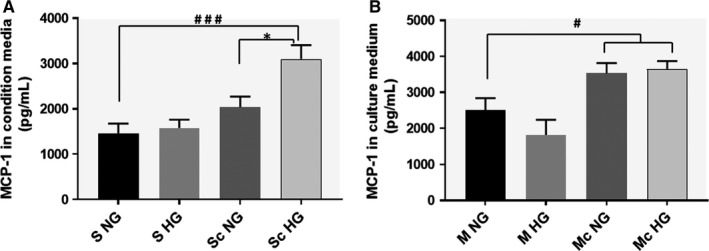
SMC‐MAC cross talk in high glucose conditions induces a significant increased level of MCP‐1 released in the conditioned medium, both in SMC (A) and MAC (B). The MCP‐1 released by macrophages was quantified in the conditioned media collected from the upper insert (of trans‐well system), while the MCP‐1 released from SMC was quantified in the conditioned media collected from the bottom chamber. As controls, MCP‐1 was quantified in the conditioned media collected from macrophages or SMC grown independently (no co‐culture) in normal or high glucose conditions. Note that consequent to the cell co‐culture, MCP‐1 is significantly released by SMC after cross‐talk with MAC in HG vs NG conditions (A). n = 4, *P < .05, ^#^ *P* < .05, ^###^ *P* < .001

It was reported that the interaction between monocytes and SMC by soluble factors synergistically enhanced MCP‐1 production 20 and we have shown also that communication between MAC and SMC leads to increased MCP‐1 expression in MAC.^17^ Here, we inquire whether the high‐glucose condition induces a similar phenomenon or even augment this process. We measured the MCP‐1 in the secretome collected from SMC or MAC following their cross‐talk in NG or HG conditions. Using an ELISA assay, we found that upon co‐culture in NG, the released MCP‐1 by SMC was not significantly different from that obtained in controls (individually cultured SMC in NG or HG conditions). In contrast, the SMC which were co‐cultured with MAC in HG supplemented media released a significant higher amount of MCP‐1 in the secretome, compared to control SMC (S NG, *P* = .0009), or SMC which were co‐cultured with MAC in NG (Sc NG ‐ *P* = .02, Figure 4A).

Analysis of the MAC's secretome revealed that these cells, upon co‐culture with SMC, released significantly higher levels of MCP‐1 in the culture medium in both, NG or HG conditions, as compared to controls (Figure 4B).

### 3.4 Signalling pathways that mediate MMPs expression in SMC induced by co‐culture with MAC in HG conditions

**Figure 5:**
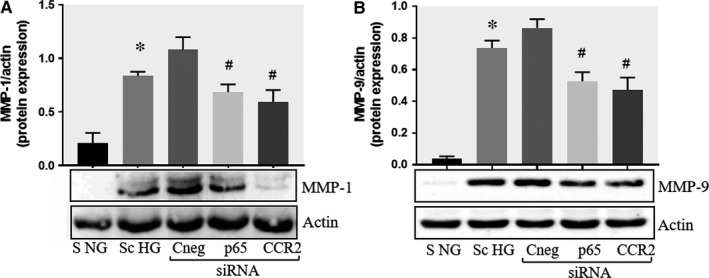
Effect of CCR2 and p65 silencing in the regulation of MMP‐1 and MMP‐9 expression in SMC. Protein expression of MMP‐1 (A) and MMP‐9 (B) determined by Western blot in control SMC (S NG), SMC which were co‐cultured with MAC in HG conditions (Sc HG), and SMC subjected to silencing of CCR‐2 and p65 and co‐cultured with MAC in HG conditions (Cneg, p65, CCR2 siRNA). Note that silencing of CCR2 or p65 decreases significantly the protein expression of MMP‐1 and MMP‐9 induced by SMC‐MAC co‐culture in high glucose conditions. n = 3, *P < .05, ^#^ *P* < .05

Previously, it was reported that binding of MCP‐1 to CCR2, triggers cell signalling and activation of NF‐kB transcription factor.^24^ Moreover, we found that both, the cell cross‐talk or HG levels activate NF‐kB in SMC.^18^, ^25^ Thus, we examined the possible role of MCP‐1 and NF‐kB in the regulation and increased expression of MMPs. To this purpose, the endogenous expression of CCR2 and p65 (NF‐kB subunit) was blocked in SMC with target‐specific siRNA, prior to SMC co‐culture with MAC. Transfection efficiency assessed by Western blot assays revealed a reduction of ~45% for CCR2 and of ~64% for p65 subunit of NF‐kB (Figure S2A,B). The results of these experiments showed that the protein expression of MMP‐1 and MMP‐9 were reduced significantly in SMC in which the CCR2 or p65 expressions were silenced. No effect on MMPs expression was detected in SMC transfected with negative control siRNA (Figure 5A,B).

### 3.5 Involvement of PKCα in the regulation of MMPs expression in SMC upon their co‐culture with MAC in HG conditions

**Figure 6:**
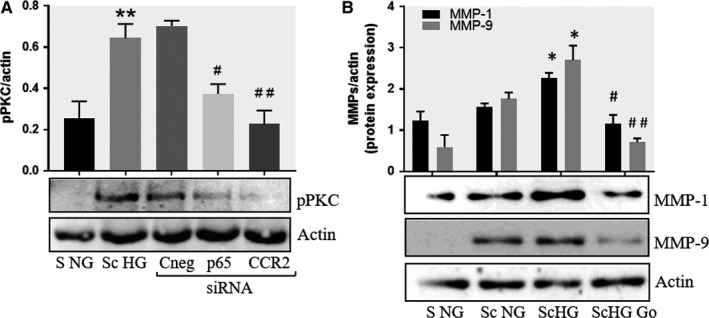
Inhibition of PKCα decreases the protein expression of MMP‐1 and MMP‐9 induced by cell cross‐talk in SMC. A, Evaluation of PKCα activation in control SMC (S NG) vs SMC previously co‐cultured with MAC in HG (Sc HG). Note that, the phosphorylated form of PKCα is significantly increased in SMC that were before exposed to MAC in HG conditions. Silencing of CCR2 or p65 decreases the phosphorylation of PKCα to control levels. B, Effect of PKCα inhibitor – Go 6976 on the MMP‐1 and MMP‐9 protein expression induced by SMC‐MAC cross‐talk. S NG – control SMC, Sc NG – SMC which were co‐cultured with MAC in normal glucose, Sc HG ‐ SMC which were co‐cultured with MAC in high glucose, Sc HGGo ‐ SMC which were co‐cultured with MAC in high glucose in presence of 1 μmol/L Go 6976. n = 3, *P < .05, **P < .01, ^#^ *P* < .05, ^##^ *P* < .01

Activation of protein kinase C (PKC) family is one of the main signalling events induced by HG concentration.^26^ To examine the possible involvement of PKC in the up‐regulation of MMP‐1 and MMP‐9 we evaluated the activation of PKCα in SMC. As resulted from Western blot experiments, the MAC‐SMC co‐culture induced a significant increase in the phosphorylation of PKCα in SMC. Interestingly, silencing of p65 or CCR2 by specific siRNA, impeded the activation of PKCα (Figure 6), suggesting a direct involvement of MCP‐1 signaling in the process. To evaluate whether PKCα is directly implicated in the MMP‐1 and MMP‐9 expression, in additional experiments, the co‐culture between MAC and SMC was done in the presence of PKCα inhibitor, Go 6976 (1 μmol/L). In this case, the protein expression of both, MMP‐1 and MMP‐9 were reduced significantly, suggesting that activation of PKCα is involved in MMP‐1 and MMP‐9 over‐expression.

### 3.6 Collagen assembly by SMCs

**Figure 7:**
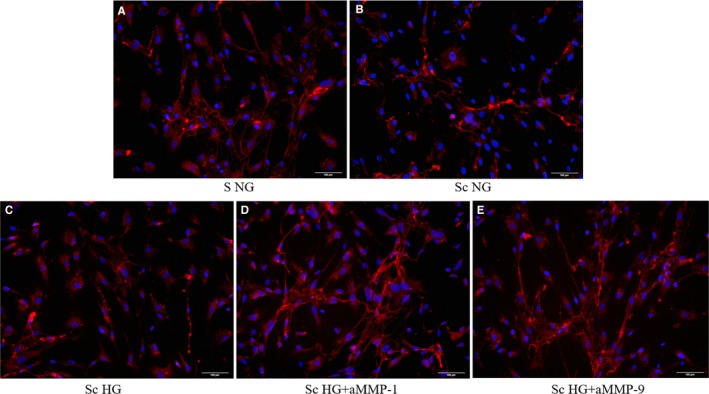
Formation of collagen fibres from soluble collagen labelled with Texas Red by SMC. A‐ control SMC cultured in normal glucose conditions (S NG), B‐ SMC after their co‐culture with MAC in normal glucose (Sc NG) or under high glucose conditions (C ‐ Sc HG), (D and E) ‐ SMC after their co‐culture with MAC in HG and pre‐incubated with blocking anti‐MMP‐1 or 9, respectively, before collagen addition; red ‐ stained collagen fibers; blue ‐ nuclei of SMC stained with Hoechst

The cross‐talk between SMC and monocytes/MAC influences SMC that turn towards a secretory phenotype, an event associated with the progression of atherosclerosis.^27^ Here, we investigated whether SMC with activated state after co‐culture with MAC, retained the ability to construct collagen fibrils from collagen precursors, a crucial process for vascular stability.^28^, ^29^ For this, Texas red‐labelled solubilized collagen was added to control SMC or to SMC after their co‐culture in NG or HG with MAC, and fibril assembly was followed by fluorescent microscopy. The results revealed that after co‐culture with MAC in NG, and even more in HG conditions, SMC diminished their capacity to assemble the collagen monomers in fibres of collagen, as shown in Figure 7B,C, respectively. Interestingly, if SMC (after their co‐culture with MAC in HG) were pre‐incubated for 30 minutes with blocking anti‐MMP‐1 or anti‐MMP‐9 and next was added Texas red‐labelled collagen, the SMC recover their capacity to assemble the collagen fibres (Figure 7D,E). Thus, we can assume that after the cross‐talk with MAC in normal, and even more pronounced in HG conditions, the capacity of SMC to form collagen fibers is impaired, and the released MMP‐1 and MMP‐9 can be involved in this process.

## 4 DISCUSSION

Despite the extraordinary effort done over the last 50 years on atherosclerosis research, little is known about the late stages of atherosclerosis, namely, what are the specific processes/ molecules that control the fine balance between the stable and instable (prone to rupture) plaques. The reason for the lack of understanding of this crucial step, could be the fact that most data come from the mouse as experimental model, which is not an appropriate model for the study of the late stages of atherogenesis. Therefore, although several mouse models exhibiting atherosclerotic plaques that are histologically similar to the human advanced lesions were developed, a consensus model for atherosclerotic plaque destabilization is still lacking.^30^ This is especially true in the case of diabetes, where the complicated atherosclerotic plaques exhibit amplified calcification, necrotic cores, monocyte and T‐cell infiltration and a higher incidence of plaque ruptures and vascular remodelling.^31^

To identify the specific processes/molecules relevant for the higher vulnerability of atherosclerotic plaque in diabetic patients, we setup an in vitro system to examine whether the cross talk between MAC and SMC in HG conditions (mimicking diabetes) affect the synthesis and enzymatic activity of MMPs, known as major players in atherosclerotic plaque vulnerability. In addition, we questioned the signalling mechanisms involved in this process.

The novel findings reported here reveal that upon cross‐talk with MAC in HG conditions, SMC exhibit: (*i*) augmented gene and protein expression of MMP‐1 and MMP‐9; (*ii*) a significant increase in the enzymatic activity of MMP‐9; (*iii*) higher levels of soluble MCP‐1 chemokine which is functionally active and involved in MMPs up‐regulation; (*iv*) activated PKCα signalling pathway that, together with NF‐kB, are responsible for MMP‐1 and MMP‐9 up‐regulation, and (*v*) impaired function of collagen assembly.

Although the exact mechanism for thinning and rupture of the atherosclerotic fibrous cap are not well‐known, some hypotheses were proposed. Since SMC are the main producers of plaque‐stabilizing fibres, ie collagens, one of the main hypotheses is that diminished number of SMC (by their apoptosis) is responsible for plaque rupture. Other hypothesis is that the plaque rupture is not necessarily the result of SMC loss, but rather is due to their switch in phenotype, from contractile to synthetic SMC.^27^ These synthetic SMC acquire increased capacity for cell proliferation, migration, and secretion of various ECM proteins, preventing rupture of the fibrous cap.^27^ However, SMC phenotypic switching may also result in different form of less‐differentiated SMC (which loose SMC markers), including macrophage‐like cells, that lose the capacity to produce matrix proteins and gain the ability to synthesize an increased number of inflammatory molecules and MMPs, which may exacerbate the plaque inflammation and damage the ECM molecules.^27^

Previous studies have shown that both, SMC apoptosis or the secretory phenotype of SMC, could be induced be different agonists, various conditions or cell‐to‐cell cross talk. Thus, recently we have reported that in culture, SMC ‐ MAC cross‐talk reduces the collagen and elastin expression and promotes neo‐angiogenesis.^9^ Here we illustrate that SMC cross‐talk with MAC in HG conditions has a synergistic effect on the expression of 2 key MMPs that function in matrix destruction, MMP‐1 and MMP‐9. Moreover, we found that the enzymatic activity of MMP‐9 released by SMC is enhanced upon their prior dialogue in culture. These results, together with the un‐modified expression of TIMP‐2 evaluated after cell cross‐talk (Figure S3), suggest that SMC‐MAC dialogue could have an enhanced deleterious effect on matrix remodelling and destruction in HG environment.

Reportedly, the level of MMP‐1 and MMP‐9 increases in association with diabetic conditions. MMP‐1 is up‐regulated in the vitreous samples collected from patients with diabetic retinopathy, compared to nondiabetic controls, and the interactions among MMP‐1, thrombin, PAR1 and VEGF might facilitate angiogenesis in these patients.^32^ In addition, it was found that HG concentration and IL‐6 synergistically augment MMP‐1 expression in U937 mononuclear phagocytes and stimulate MMP‐1 secretion in human primary monocytes.^33^ In another study, HG in association with IFN‐γ synergistically stimulate MMP‐1 expression in U937 MAC.^34^ Interestingly, in both reports, as well as in our results, the HG levels alone do not significantly affect the MMP‐1 expression, but rather acts synergistically with IL‐6,^33^ IFNN‐γ 34 or with SMC‐MAC cross‐talk (our results). In the case of MMP‐9, although it was found that serum level of MMP‐9 in patients with acute coronary syndrome with hyperglycaemia was significantly higher than those without hyperglycaemia,^35^ the source and the mechanism that explain this increase in diabetic patients were not identified. In this context, our results indicate that SMC as a possible source of increased MMPs production in accelerated vascular disorders associated with diabetes.

Regarding the mechanisms involved in SMC‐induced increased MMPs production we searched for the involvement of CCR2 ‐ PKC ‐ NF‐kB pathway, as triggered by MCP‐1. We report here that the release of MCP‐1 by SMC increase significantly after their co‐culture with MAC in HG conditions. Previous work demonstrated that mutation of MCP‐1 prevents vulnerable plaques from rupture in rabbits independent of serum lipid levels.^36^ Therefore, we supposed that, upon binding to its main receptor CCR2, MCP‐1 could trigger MMPs induction in SMC. As it is known, binding of MCP‐1 to CCR2 may promotes protein kinase C (PKCα) phosphorylation and ultimately cause NF‐κB activation.^37^ The reduction in MMP‐1 or MMP‐9 protein levels by CCR2 and p65 subunit knockdown experiments, realized before cell co‐culture, confirmed our hypothesis, that the MCP‐1/CCR2 pathway is implicated in the process. As shown in the Figure 5, the MMP‐1 and MMP‐9 protein expression was not reduced to the baseline levels by CCR2 and p65 silencing, suggesting that additional mechanisms can be involved in the MMPs regulation. Previously, it was demonstrated that AP‐1 transcription factor has a pivotal role in MMPs (including MMP‐1 and ‐9) transcription in many cells (reviewed in 38). Moreover, the mitogen activated protein kinases (MAPK), the signal transducers and activators of transcription (STAT) and the Smad family of proteins are also involved in the activation of MMPs transcription.^38^

We also found an increased phosphorylation of PKCα in SMC after their co‐culture with MAC in HG conditions, which is impeded by silencing of CCR2. This result reinforces the above hypothesis on the role of MCP‐1/CCR2 in the activation of PKC pathway. Moreover, when cell co‐culture in HG where realized in presence of a selective inhibitor for PKCα (GO 6976), the protein expression of MMP‐1 and MMP‐9 where significantly reduced, confirming the involvement of PKCα in their up‐regulation. Our data corroborate and extend previous reports that acknowledge the participation of PKC signalling in MMPs over‐expression in PMA‐activated monocytes 39, 40 and SMC exposed to cytokines.^41^

For a long time, it was believed that collagens (especially collagen type I) have the capacity to self‐assemble into fibrils in the absence of cellular interactions.^42^ However, in the last decades, it was demonstrated that the polymerization process of type I collagen is actually dependent on cellular mechanisms or interactions.^28^ Our results demonstrate that indeed SMC have this ability, except that after co‐culture with MAC, SMC lose their capacity of collagen assembly, in particular in HG conditions. Interestingly, SMC recover their function to assemble the collagen fibres when the ‐MMP‐1 and ‐9 are blocked (Figure 7D,E), revealing that these MMPs produced and released by cell co‐culture are functional active and degrade the collagen fibres.

Vascular SMC in advanced atherosclerotic lesions are generally regarded as having athero‐protective plaque‐stabilizing properties. However, the change in phenotype, namely the switch of SMC towards an inflammatory/secretory phenotype, in conjunction with the production of high levels of MMPs and impaired capacity for collagen assembly, propose SMC as a destabilization factor, involved rather in plaque rupture than in plaque stabilization. Thus, SMC are an attractive target and efforts should be focused on these key cells involved in the fine balance between stable and vulnerable plaques.

## 5 STUDY LIMITATION

In this study, the majority of results were obtained using co‐culture between human aortic SMC and the MAC differentiated from human monocytic cell line THP, in normal or HG conditions. Although some results were confirmed with MAC derived from control or diabetic patients, further studies employing human atherosclerotic plaques biopsies isolated from diabetic patients will be necessary to strengthen the significance of our novel observations.

Taken together, these data highlight the important role of SMC that upon communication with MAC in HG, produce a significant increase in active matrix degradation enzymes, event which along with reduced collagen assembly function can count for the high plaque vulnerability found in diabetic patients.

## CONFLICT OF INTEREST

All authors state that they have no conflict of interests.
