# Supplementary figures and images for: The expression of MMP‐1 and MMP‐9 is up‐regulated by smooth muscle cells after their cross‐talk with macrophages in high glucose conditions
Source: J Cell Mol Med. 2018 Jul 10;22(9):4366–76. doi: 10.1111/jcmm.13728 (PMC6111860; doi:10.1111/jcmm.13728)

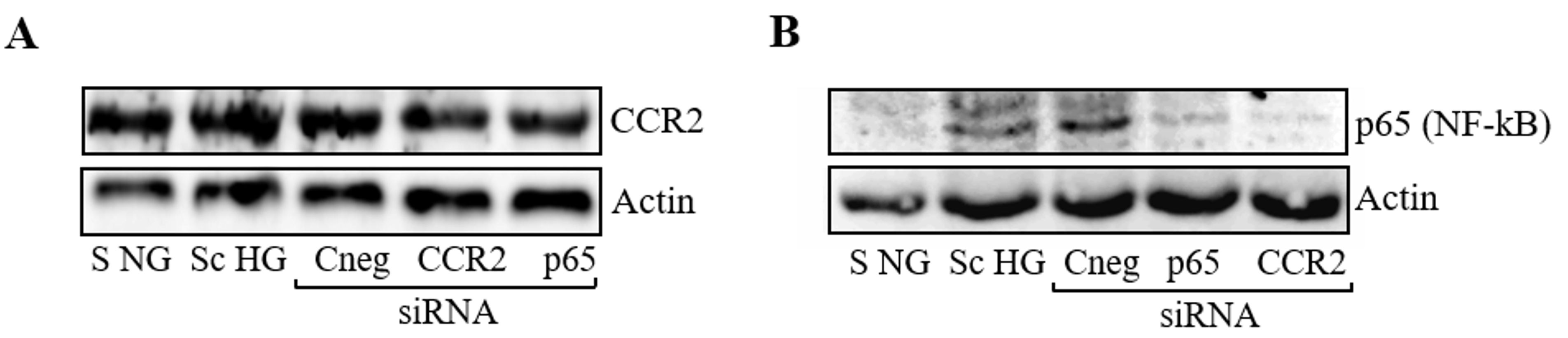

Supplement: Supplementary file 2 [file JCMM-22-4366-s002.tif]

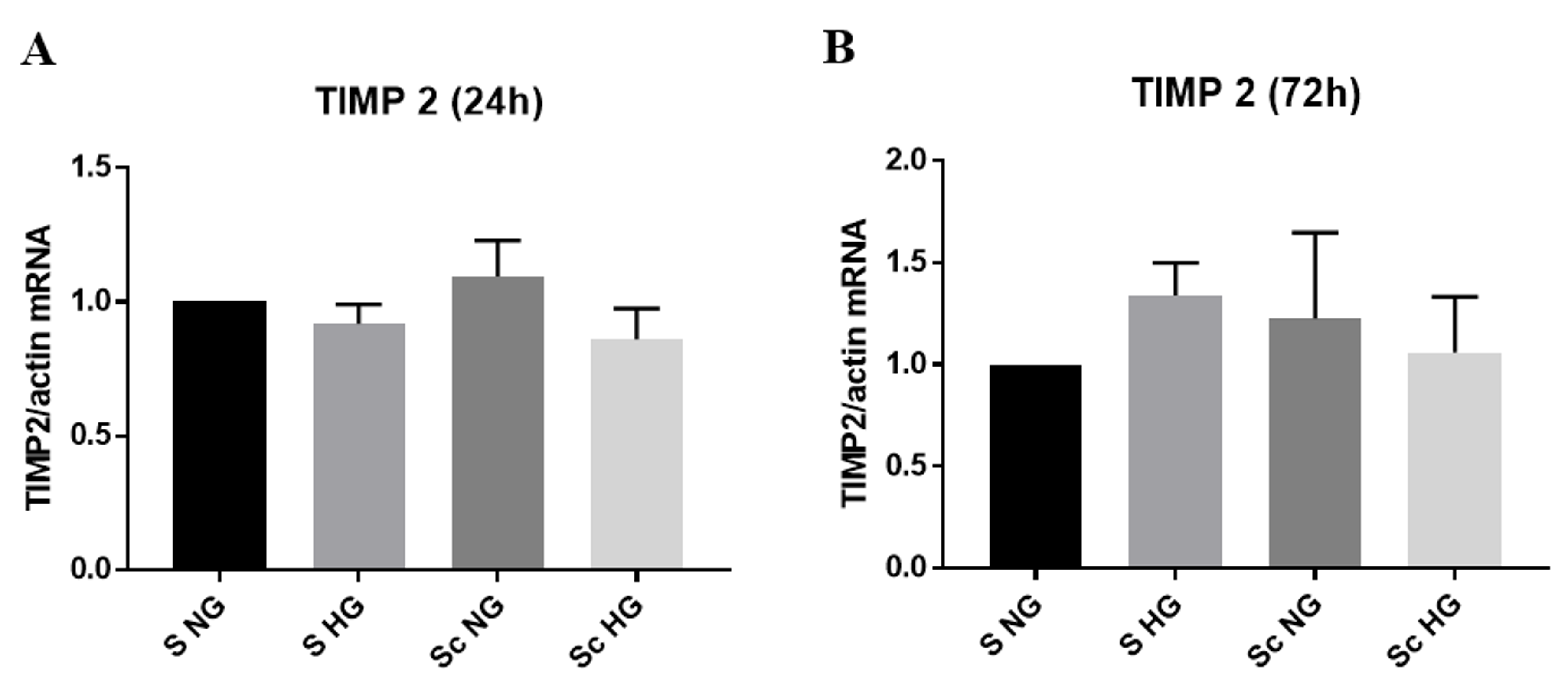

Supplement: Supplementary file 3 [file JCMM-22-4366-s003.tif]
